# A Reference Genome Sequence for the European Silver Fir (*Abies alba* Mill.): A Community-Generated Genomic Resource

**DOI:** 10.1534/g3.119.400083

**Published:** 2019-06-26

**Authors:** Elena Mosca, Fernando Cruz, Jèssica Gómez-Garrido, Luca Bianco, Christian Rellstab, Sabine Brodbeck, Katalin Csilléry, Bruno Fady, Matthias Fladung, Barbara Fussi, Dušan Gömöry, Santiago C. González-Martínez, Delphine Grivet, Marta Gut, Ole Kim Hansen, Katrin Heer, Zeki Kaya, Konstantin V. Krutovsky, Birgit Kersten, Sascha Liepelt, Lars Opgenoorth, Christoph Sperisen, Kristian K. Ullrich, Giovanni G. Vendramin, Marjana Westergren, Birgit Ziegenhagen, Tyler Alioto, Felix Gugerli, Berthold Heinze, Maria Höhn, Michela Troggio, David B. Neale

**Affiliations:** * C3A - Centro Agricoltura Alimenti Ambiente, University of Trento, via E. Mach 1, 38010 S. Michele a/Adige (TN), Italy; † CNAG-CRG, Centre for Genomic Regulation (CRG), The Barcelona Institute of Science and Technology, BaldiriReixac 4, 08028 Barcelona, Spain; ‡ Fondazione Edmund Mach, Via Mach 1, 38010 S. Michele a/Adige (TN), Italy; § Swiss Federal Research Institute WSL, Zürcherstrasse 111, 8903 Birmensdorf, Switzerland; †† University of Zürich, Department of Evolutionary Biology and Environmental Studies, Winterthurerstrasse 190, CH-8057 Zurich; ‡‡ Institut National de la Recherche Agronomique (INRA), Unité de Recherche Ecologie des Forêts Méditerranéennes (URFM), Site Agroparc, Domaine Saint Paul, 84914 Avignon, France; §§ Thünen-Institute of Forest Genetics, Sieker Landstr, 2, 22927 Grosshansdorf, Germany; ** Bavarian Office for Forest Seeding and Planting (ASP), Applied Forest Genetics, Forstamtsplatz 1, 83317 Teisendorf, Germany; ††† Technical University in Zvolen, TG Masaryka 24, 96053 Zvolen, Slovakia; ‡‡‡ Institut National de la Recherche Agronomique (INRA), UMR1202 Biodiversity, Genes & Communities (BIOGECO), University of Bordeaux, 69, route d’Arcachon, 33610 Cestas, France; §§§ INIA Forest Research Centre, Carretera de la Coruña km 7.5, 28040 Madrid, Spain; *** Universitat Pompeu Fabra (UPF), Plaça de la Mercè, 10, 08002 Barcelona, Spain; †††† Department of Geosciences and Natural Resource Management (IGN), University of Copenhagen, Rolighedsvej 23, 1958 Frederiksberg C, Denmark; ‡‡‡‡ Philipps-Universität Marburg, Faculty of Biology (PUM), Karl-von-Frisch-Str. 8, 35032 Marburg, Germany; §§§§ Department of Biological Sciences (METU), Middle East Technical University, 06800 Çankaya/Ankara, Turkey; **** Department of Forest Genetics and Forest Tree Breeding, Georg-August University of Göttingen, Büsgenweg 2, 37077 Göttingen, Germany; ††††† Laboratory of Population Genetics, Vavilov Institute of General Genetics, Russian Academy of Sciences, Gubkina Str. 3, 11991 Moscow, Russia; ‡‡‡‡‡ Laboratory of Forest Genomics, Genome Research and Education Center, Institute of Fundamental Biology and Biotechnology, Siberian Federal University, 50a/2 Akademgorodok, 660036 Krasnoyarsk, Russia; §§§§§§ Max Planck Institute for Evolutionary Biology, Department for Evolutionary Genetics (MPI), August Thienemann Str. 2, 24306 Ploen, Germany; ***** Institute of Biosciences and BioResources, National Research Council, Via Madonna del Piano 10,50019 Sesto Fiorentino (Firenze), Italy; †††††† Slovenian Forestry Institute (SFI), Gozdarskiinštitut Slovenije), Večna pot 2, 1000 Ljubljana, Slovenia; ‡‡‡‡‡‡ Federal Research and Training Centre for Forests, Natural Hazards and Landscape (BFW), Seckendorff-Gudent Weg 8, 1130 Wien, Austria; §§§§§§ Faculty of Horticultural Science, Department of Botany (SZIU/FHS), Szent Istvan University, 1118 Budapest, Hungary; ****** Department of Plant Sciences, University of California at Davis (UCD), Davis 95616

**Keywords:** *Abies alba*, annotation, conifer genome, genome assembly, chloroplast genome

## Abstract

Silver fir (*Abies alba* Mill.) is a keystone conifer of European montane forest ecosystems that has experienced large fluctuations in population size during during the Quaternary and, more recently, due to land-use change. To forecast the species’ future distribution and survival, it is important to investigate the genetic basis of adaptation to environmental change, notably to extreme events. For this purpose, we here provide a first draft genome assembly and annotation of the silver fir genome, established through a community-based initiative. DNA obtained from haploid megagametophyte and diploid needle tissue was used to construct and sequence Illumina paired-end and mate-pair libraries, respectively, to high depth. The assembled *A. alba* genome sequence accounted for over 37 million scaffolds corresponding to 18.16 Gb, with a scaffold N50 of 14,051 bp. Despite the fragmented nature of the assembly, a total of 50,757 full-length genes were functionally annotated in the nuclear genome. The chloroplast genome was also assembled into a single scaffold (120,908 bp) that shows a high collinearity with both the *A. koreana* and *A. sibirica* complete chloroplast genomes. This first genome assembly of silver fir is an important genomic resource that is now publicly available in support of a new generation of research. By genome-enabling this important conifer, this resource will open the gate for new research and more precise genetic monitoring of European silver fir forests.

Conifers represent the dominant trees in some temperate and all boreal ecosystems and have important economic value. They face the effects of climate change, with an increase in temperature and lower precipitation, and increased frequency of extreme events, to which some species may be unable to quickly adapt. Silver fir (*Abies alba* Mill.) is a keystone conifer of European montane forest ecosystems, which is dominant in cool areas of the temperate zone (Ellenberg 2009). Its distribution ranges from the Pyrenees to the Alps and the Carpathians where it reaches its easternmost range edge (Figure S1). Growing interest in silver fir has emerged because of its potential vulnerability to climate change (Cailleret *et al.* 2014), which could change conditions for its sustainable use and its economic value. In turn, this species is more drought-resistant than other important species for timber production, such as Norway spruce (Vitali *et al.* 2017), which could turn out to be beneficial under the expected increase in extended future drought periods.

Several studies investigated the environmental effect on silver fir genetic diversity across the Italian Alps, showing the association between its genetic diversity and seasonal minimum temperature (Mosca *et al.* 2012) as well as between genetic diversity and both temperature and soil type (Mosca *et al.* 2014). Recent studies confirmed the environmental effect on the species’ local adaptation, which was shaped by winter drought in marginal populations (Roschanski *et al.* 2016); while in common gardens, selection on height was driven by thermal stability (Csilléry *et al.* 2018). Another study confirmed the importance of the Apennines as a refugium of genetic diversity (Piotti *et al.* 2017). All these studies were based on a modest number of genetic markers (several hundred of single-nucleotide polymorphisms, SNPs, or tens of simple sequence repeats, SSRs, also called microsatellites) due to the lack of genomic resources.

Conifer genomes are very large, ranging from 4 to 35 giga base pairs (Gb) (Bennett and Leitch 2011; Grotkopp *et al.* 2004; Zonneveld 2012), but their gene content is similar to that of other vascular plants (Leitch *et al.* 2005). Conifer genomic resources have grown in recent years due to the application of high throughput sequencing technologies (Reuter *et al.* 2015). To date, a few conifer genomes have been fully sequenced, including: *Picea abies* (L.) Karst (Nystedt *et al.* 2013), *Picea glauca* (Moench) Voss (Warren *et al.* 2015), *Pinus taeda* L. (Neale *et al.* 2014), *Pinus lambertiana* Dougl. (Stevens *et al.* 2016), *Pseudotsuga menziesii* (Mirb.) Franco (Neale *et al.* 2017) and *Larix sibirica* Ledeb (Kuzmin *et al.* 2019).

The present research is part of the Silver Fir Genome Project, which is a community effort within the Alpine Forest Genomics Network (AForGeN, IUFRO WP 2.04.11, https://www.aforgen.org). This network was established in 2011 with the intent to facilitate information exchange and collaboration among researchers interested in studying adaptation in alpine forest ecosystems to climate change, using landscape genomics approaches (Neale *et al.* 2013). Within this researcher community arose the idea to launch the genome project of an important subalpine conifer species. The genome sequencing was financed by a bottom-up approach among partners (https://sfgp.faculty.ucdavis.edu/).

The aim of this project was to sequence and assemble the silver fir genome, and to compare this resource with other available conifer genomes. This study provides additional information on the *Abies* chloroplast genome in relation to closely related taxa. A long-term perspective is to identify gene regions involved in drought resistance and late flushing, which are traits found to be important in Mediterranean firs (George *et al.* 2015).

## Materials and Methods

### Reference tree for genome sequencing

Tissue samples for sequencing were obtained from an adult silver fir tree (AA_WSL01) located in a public forest next to the institute of WSL Birmensdorf, Switzerland (47.3624°N, 8.4536°E). Seeds were collected directly from the selected tree in November 2016, dried at ambient temperature and stored at -5°. Fresh needles were harvested shortly after flushing in May 2017. A multilocus SNP analysis across 19 Swiss populations placed the sampled tree mainly in the genetic cluster of other Swiss plateau populations, with some ancestry similar to populations in the Jura Mountains and in the Northern Alps (Figure S2).

### DNA preparation

#### Haploid megagametophyte DNA isolation for paired-end (PE) sequencing:

Seeds of the reference tree were incubated in tap water for 24 h at room temperature. Seeds were dissected in a sterile 0.9% sodium-chloride solution under a stereo lens in an environment cleaned with bleach, using micro scissors and forceps. The diploid nucellar and integument tissues were carefully removed. The retained megagametophyte tissue was rinsed with fresh sterile 0.9% sodium-chloride solution, immediately transferred to a 2 mL Eppendorf tube and stored at -80°. Megagametophyte tissue was lyophilized for 16 h prior to extraction and homogenized for 30 s using a mixer mill (Retsch MM 300, Haan, Germany). DNA extraction was performed with a customized sbeadex kit (LGC Genomics, Berlin, Germany), which included chemicals and reagents as described below. 500 µL LP-PVP, 5 µL Protease, 1 µL RNAse and 20 µL debris capture beads were added as lysis buffer to the ground tissue and the mix was incubated at 50° and 350 rounds per minute (rpm) in a heating block for 30 min. After brief centrifugation, 400 µL cleared lysate was added to 400 µL binding buffer SB and 10 µL sbeadex beads. After 15 min binding at room temperature with shaking at 850 rpm, magnetic beads were collected on a magnetic stand for 2 min, and the supernatant was discarded completely. Beads were successively washed with the following buffers: 400 µL BN1, 400 µL TN1, 400 µL TN2, and 400 µL PN2. Washing time was 7 min for all four steps, with shaking at 850 rpm, followed by a short spin, 2 min of bead collection on a magnetic stand, and careful discarding of wash buffer. DNA was finally eluted in 100 µL elution buffer AMP at 60° and 850 rpm on a heating block for 10 min. After a short spin and 3 min of magnetic bead collection on a magnetic stand, DNA was transferred into a new tube, centrifuged at 21,000 × g for 2 min, and transferred without pellet into a new tube.

DNA concentration was measured using the QuantiFluor dsDNA System (Promega, Madison, WI, USA). 260/280 and 260/230 ratios were measured using a Nanodrop 1000 (Thermo Fisher Scientific, Waltham, MA, USA; Table S1 Supplemental Information), and DNA integrity was visualized by running 5 µL of DNA on a 1% agarose gel. Nuclear microsatellites were used to test for the contamination of the haploid maternal DNA with diploid DNA deriving from the surrounding tissue and to confirm the presence of only one maternal haplotype (Table S2A). Because different megagametophytes from the same tree represent different haplotypes, only one DNA sample from a single megagametophyte with high DNA quality (260/280 ratio: 1.83, 260/230 ratio: 1.75) and quantity (3.7 µg at 41 ng/µL; Table S1) was used by CNAG-CRG for PE library preparation and sequencing.

#### Diploid needle DNA isolation for mate-pair (MP) sequencing:

Young, bright green needles of the reference tree were collected, frozen at -80° and lyophilized for 24 h. For DNA extraction, 25 mg of tissue were ground in a 2 mL Eppendorf tube with two steel balls (d = 3.1 mm) for 1.5 min, using a mixer mill MM300 (Retsch). DNA was extracted with the Dneasy Plant Mini Kit (Qiagen, Hilden, Germany), starting with 600 µL AP1, 1 µL RNAse and 1 µL DX reagent. Then, DNA extraction was carried out according to the manufacturer’s protocol, with an additional washing step with washing buffer AW2. DNA was eluted in 2x 100 µL nuclease-free water. DNA concentration was measured using QuantiFluor dsDNA System (Promega), 260/280 and 260/230 ratios were measured using a Nanodrop 1000 (ThermoFisher), and DNA integrity was visualized by running 0.6 µL of DNA on a 1% agarose gel. DNA samples were checked for contamination again using nuclear microsatellite markers (Table S2A), and one sample (24.5µg at 136 ng/µL; Table S1) was used for MP sequencing.

### Sequencing

#### Whole-genome sequencing (WGS) library preparation and sequencing:

Haploid DNA from the single megagametophyte was used to construct three 300 bp-insert paired-end libraries at the CNAG-CRG Sequencing Unit. The short-insert PE libraries for the whole-genome sequencing were prepared with KAPA HyperPrep kit (Roche-Kapa Biosystems) with some modifications. In short, 1.0 µg of genomic DNA was sheared on a Covaris LE220 (Covaris Woburn, Massachusetts, USA) in order to reach fragment sizes of ∼500 bp. The fragmented DNA was further size-selected for fragment sizes of 220-550 bp with AMPure XP beads (Agencourt, Beckman Coulter). The size-selected genomic DNA fragments were end-repaired, adenylated and ligated to Illumina sequencing compatible indexed paired-end adaptors (NEXTflex DNA Barcodes). The adaptor-modified end library was size-selected and purified with AMPure XP beads to eliminate any non-ligated adapters. The ligation product was split into three samples and in three separate reactions enriched with 12 PCR cycles and then validated on an Agilent 2100 Bioanalyzer with the DNA 7500 assay (Agilent) for size and quantity. The resulting libraries had estimated fragment sizes of 304 bp, 307 bp and 311 bp. These are referred to as PE300-1, PE300-2, and PE300-3 in Table 1.

**TABLE 1:** Summary of the raw data for Illumina paired-end (PE) and mate-pair (MP) libraries for whole-genome sequencing of *Abies alba*

Library	Read length (bp)	Insert size (kb)	Mean fragment size (bp)	Read Pairs (million)	Yield (Mb)	Coverage	Avg. Phix Error R1 (%)	Avg. Phix Error R2 (%)
PE300-1	2 x 151	—	304	3,274	989,029	57.103	0.646	0.908
PE300-2	2 x 151	—	307	1,886	569,617	32.888	0.883	1.126
PE300-3	2 x 151	—	312	1,066	322,181	18.602	0.768	1.081
MP1500	2 x 101	1.5	—	1,255	253,529	14.638	0.214	0.32
MP3000	2 x 101	3	—	1,277	257,985	14.895	0.214	0.32
MP8000	2 x 101	8	—	1,255	253,590	14.641	0.214	0.32
Total PE				6,226	1,880,827	108.593		
Total MP				3,787	765,104	44.175		

All three libraries were sequenced in equal proportions on HiSeq 4000 (Illumina, Inc, San Diego, California, USA) in paired-end mode with a read length of 2 × 151 bp using a HiSeq 4000 PE Cluster kit sequencing flow cell, following the manufacturer’s protocol. Image analysis, base calling and quality scoring of the run were processed using the manufacturer’s software Real Time Analysis (RTA 2.7.6) and followed by generation of FASTQ sequence files by CASAVA.

#### Mate-pair library preparation and sequencing:

DNA extracted from diploid needles was used to build three mate-pair (MP) libraries of increasing insert size of 1,500 bp, 3,000 bp and 8,000 bp (MP1500, MP3000, MP8000). Libraries were prepared using the Nextera Mate Pair Library Prep Kit (Illumina) using the gel-plus protocol selecting for three different distribution sizes according to the manufacturer’s instructions. After fragmentation, bands of 1.5, 3 and 8 Kb were selected for circularization. The following amounts of size-selected DNA were used for the circularization reaction: 270 ng (1.5 Kb), 285 ng (3 Kb), and 97.4 ng (8 Kb).

All three MP libraries were sequenced on HiSeq2000 (Illumina) in paired-end mode with a read length of 2 × 101 bp using TruSeq SBS Kit v4. Image analysis, base calling and quality scoring of the run were processed using the manufacturer’s software Real Time Analysis (RTA 1.18.66.3) and followed by generation of FASTQ sequence files by CASAVA.

### Assembly

#### Genome assembly:

Given the nearly equivalent estimated fragment sizes, the reads from the three PE libraries (PE300-1, PE300-2, and PE300-3) were joined into one library for assembly and collectively referred to as PE300. Before assembling the genome, its size and its complexity were evaluated using *k*-mer analyses. Jellyfish v2.2.0 (Marçais and Kingsford 2011) was run on the sequence reads of this PE library to obtain the distribution of 17 *k*-mers. SGA preqc (Simpson and Durbin 2011; Simpson 2014) was then used to estimate the mean fragment size and standard deviation of the PE300 library.

First, an initial assembly of the PE300 reads was performed with MaSuRCA v3.2.2 (Zimin *et al.* 2013) using default parameters, choosing SOAPdenovo for faster contig and light scaffold assembly. A *k*-mer of 105 was chosen by MaSuRCA for *de Bruijn* graph construction. The initial assembly was run for 33 days on a single 48-core node (4 Intel Xeon CPU E7-4830 v3 at 2.10GHz and 2TB of RAM) and with a maximum memory usage of 1.22 TB.

Second, the PE300 and the three MP libraries (MP1500, MP3000, MP8000) were used to scaffold the initial assembly with BESSTv2.2.5 (Sahlin *et al.* 2014). It was run with options *–separate_repeats*, -*K=*105 *–max_contig_overlap* = 115 and *–k* = 466. Briefly, –K specifies the *k*-mer size used in the *de Bruijn* graph for the input assembly to be scaffolded. As 90% of the input “contigs” were longer than 115 bp, this length was selected, instead of the default value of 200 bp, as the maximum identical overlap to search (*k*). Given the fragmented input assembly, the idea was to avoid using contigs smaller than the original genomic fragment. Therefore, the contig size threshold for scaffolding was set to 466 bp, 10 bp greater than the mean (294) plus two times the standard deviation (81) of the PE300 fragment size as estimated by mapping. The scaffolded genome assembly is referred to as ABAL 1.0. Moreover, an analysis of the spectra copy number (KAT; Mapleson *et al.* 2017) with default k = 27 was done to compare the PE300 library to the assembly. The KAT program is often used to compare the proportion of k-mers present in the reads that end up in the final assemblies. It shows how much the genome architecture agrees with the final assembly.

#### Chloroplast genome assembly and annotation:

All of the 100 bp reads from the MP1500 library (the library with the tightest size distribution and highest complexity) were mapped to the closest complete reference chloroplast sequence available in NCBI, *i.e.*, from *Abies koreana* (NC_026892.1, Yi *et al.* 2015), using BWAmem (Li and Durbin 2010) in paired mode and option –M to discard short split mappings. The mapped reads were then extracted from the alignment using BAM2FASTQ v1.1.0 (Alpha GSLaH). Both the linker sequence and the Nextera adapters present in the MP sequences were removed with Cutadapt (Martin 2011). Finally, they were reversed-complemented in order to obtain an artificial PE library with insert size of 1,387 ± 327 bp.

The FAST-PLAST pipeline was run producing SPAdes (Bankevich *et al.* 2012) assemblies using a range of *k*-mers (55, 69, 87). Afterward, Ragout (Kolmogorov *et al.* 2014) was used to obtain a reference-assisted assembly. In this case, *A. sibirica* (NC_035067.1) was used as chloroplast reference to place and orient all the *A. alba* contigs. Finally, Gapfiller (Boetzer and Pirovano 2012) was used to close gaps in the chloroplast genome. The DNA diff module—from MUMMER 3.22 package (Kurtz *et al.* 2004)—was run to compare the intermediate Spases assembly with the *A. koreana* (NC_026892.1) and *A. sibirica* (NC_035067.1) complete chloroplast sequences. Finally, the annotation of the chloroplast was carried out with DOGMA (Wyman *et al.* 2004).

#### Gene completeness:

The final nuclear assembly was evaluated for gene completeness using CEGMA v2.5 (Parra *et al.* 2007), which searches for 248 ultra-conserved core eukaryotic genes (CEGs), and BUSCO v3.0.2 (Simão *et al.* 2015), using 956 single-copy orthologs from plants (BUSCO v1 plantae database).

To obtain a more comprehensive estimate of genes present in the genome assembly, the STAR software package (Domin and Gingeras 2015) was used to map the genome assembly with the silver fir RNA-seq produced by Roschanski *et al.* (2013) (GenBank accession numbers JV134525– JV157085) as well as 12 transcriptomes originating from Mont Ventoux (France) and the Black Forest (District Oberharmersbach, Germany), as reported in Roschanski *et al.* (2013) and available in the Dryad Digital Repository (Roschanski *et al.* 2015; 2016). In addition, the transcripts from *P. taeda* (Zimin *et al.* 2014) were aligned to the genome using GMAP with default options (Wu *et al.* 2016).

### Annotation

#### Protein-coding gene annotation:

Repeats were identified, annotated and masked in the silver fir genome assembly following three sequential steps. First, RepeatMasker v4.0.6 (http://www.repeatmasker.org) was run using the Pinaceae-specific repeat library included in the RepeatMasker release. Then, repeats annotated in *P. taeda* and *P. menziesii* were used in a second run of RepeatMasker. Finally, *Abies alba*-specific repeats were detected with RepeatModeler and masked with RepeatMasker.

An annotation of the genes present in the assembly was further obtained by combining transcript alignments, protein alignments and *ab initio* gene predictions as follows. The RNAseq reads mentioned above (JV134525– JV157085 in Roschanski *et al.* 2013; 2015; 2016) were aligned to the genome using STAR v2.5.4a (Dobin *et al.* 2013) with default options, and then transcript models were generated from Stringtie (Pertea *et al.* 2015) also with default options. The resulting models were given to PASA v2.2.0 (Haas *et al.* 2008) together with 2,806 *A. alba* Expressed Sequence Tags (ESTs) downloaded from NCBI on January 31^st^, 2018. Next, the TransDecoder program, which is part of the PASA package, was used to detect coding regions in the PASA assemblies. A BLASTp (Altschul *et al.* 1990) search was performed on the Transdecoder predictions against the Swiss-Prot database (The UniProt Consortium 2018). Sequences with a complete Open Reading Frame (ORF), a BLAST hit against Swiss-Prot (E-value < 1e-9), and not hitting any repeat were considered as potential candidates to train gene predictors. Of this list, the 500 sequences whose length differed the least from the length of their BLAST target were selected as the best candidate genes and used to train the parameters for three gene predictors: GeneID v1.4 (Parra *et al.* 2000), Augustus v3.2.3 (Stanke *et al.* 2006) and Glimmer (Majoros *et al.* 2004). These three gene predictors as well as GeneMark v2.3e (Lomsadze *et al.* 2014), which run in a self-trained mode, were then run on the repeat-masked ABAL 1.0 assembly. Finally, an extra run of each GeneID, Augustus and GeneMark was performed using intron data extracted from the RNAseq mappings.

The complete Pinaceae protein sets present in PLAZA (https://bioinformatics.psb.ugent.be/plaza/versions/gymno-plaza/) in January 2018, were aligned to the repeat-masked genome using exonerate v2.4.7 (Slater and Birney 2005). Moreover, all the data described above were provided as input to Evidence Modeler v1.1.1 (Haas *et al.* 2008) and combined into consensus coding sequence (CDS) models. These models were then updated with UTRs and alternative splice isoforms with two rounds of PASA updates.

To remove the potential presence of some bacterial genes in the genome annotation, a protein-based bacterial decontamination procedure was performed on the assembly and annotation. This process utilizes a BLASTp search of the annotated proteins against the bacterial non-redundant protein database from NCBI to detect genes likely to belong to bacteria. All the scaffolds containing more than 50% of bacterial genes and without conifer-specific repeats and RNAseq mappings were removed from the assembly, resulting in the final assembly ABAL 1.1.

Finally, to check for the presence of the chloroplast genome in the nuclear genome assembly, the chloroplast assembly was mapped to ABAL 1.1 using Minimap2 (Li 2018) with the parameter–asm10. Sixty-six unique mappings longer than 1 Kb were found in the assembly (the longest being 18.49 Kb) but they did not meet the threshold of at least 70% matches. Therefore, these regions were considered as nuclear sequence homologous to chloroplast and were kept in the ABAL 1.1 assembly.

The proteins resulting from the structural annotation process described above were functionally annotated using the Blast2GO v4.1 pipeline (Conesa *et al.* 2005) with default parameters. The annotated proteins were first scanned for InterProScan patterns and profiles. Next, a BLASTp search against the NCBI RefSeq database (Uniprot and Swissprot databases) was performed, inheriting the functional annotations of the top-20 BLAST hits with an e-value < 1e-06. Finally, Blast2GO produced a consensus annotation.

In addition, the software CateGOrize (Zhi-Liang *et al.* 2008) was run to assign all genes to the main Gene Ontology (GO) categories. The software provides the count and percentage of the GO term assigned in each category. Two classification lists (slim2 and myclass2) were used in the analysis. The slim2 list is a subset of gene ontology terms (https://www.animalgenome.org/tools/catego/.goslim/GO_slim2). Myclass2 classification list is based on slim2 with 50 additional GO term categories (Table S3). The percentages across the two classification lists were visualized using the *geom_col* function of the “ggplot” package in R CRAN.

#### Comparison With other conifers:

The summary statistics on the annotated genes were computed using a custom Python script (Supplementary Material 2). The same script was applied to calculate the length of exons, introns and genes in other conifer assemblies, such as *P. abies* v1.0, *P. glauca* v3.0, *P. lambertiana* v1.5, *P. taeda* v2.0 and *P. menziesii* v1.5. The distributions of the exon, intron, gene and transcript lengths across the genome were visualized using the *violinBy* function of the “psych” package in R CRAN (R version 3.3.3; 2017-03-06).

### Data availability

The silver fir genome assembly ABAL 1.1 is available in the TreeGenes Database under https://treegenesdb.org/FTP/Genomes/Abal/. The following data are listed in the supplementary tables: the estimation of DNA concentration (Table S1), the multi-locus microsatellite genotypes of the megagametophyte and needle tissue used for sequencing (Table S2A), the genotype of AA_WSL_01 for the SNP loci (Table S2B), the Gene ontology (GO) term categories used to count the GO terms in *A. alba* (Table S3), the *A. alba* genome annotation statistics (Table S4), the intron and exon statistics for *A. alba* and *Pseudotsuga menziesii* (Table S5), and the count and percentage of the GO terms (Table S6). The following supplementary figures are included in the supplementary file: *Abies alba* distribution map (Figure S1), the location of the sampled tree AA_WSL01 along with the location of the other 19 Swiss *A. abies* populations (Figure S2), plot for the comparison between *Abies* chloroplast (Figure S3), boxplots of the gene distribution lengths in *A. alba* (Figure S4) and in other conifers (Figure S5), distribution of the most abundant GO terms. The Python script for the genome summary statistics is presented in Supplementary Material 2. Supplemental material available at FigShare: https://doi.org/10.25387/g3.7706717.

## Results and Discussion

### Genome sequencing and genome size estimation

The sequencing strategy used in this project combined Illumina PE and MP libraries following a protocol similar to that used to sequence other conifer genomes (Neale *et al.* 2017). PE and MP sequencing produced a total of 1,880,827 and 765,104 Mb, respectively (Table 1). The mean fragment size of the PE300 library estimated using *SGA preqc* was 294 bp with a standard deviation of 81 bp.

**Figure 1:**
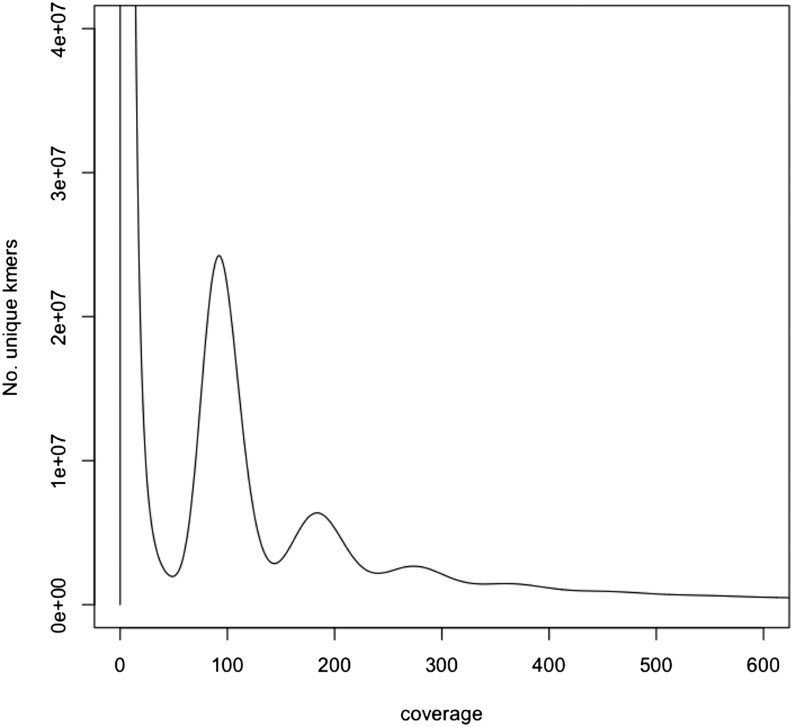
Distribution of 17-mers in the whole-genome sequence of *Abies alba* using raw paired-end (PE) 2 × 151 bp reads generated from the PE300 library with 300 bp long fragment inserts and estimated with Jellyfish 2.2.0 (Marçais and Kingsford 2011). The high peak at very low depths is caused by sequencing errors.

The estimate of the silver fir genome size, using the distribution of 17-mers (Figure 1) is 17.36 Gb, slightly higher than previous empirical estimates of the haploid C-value of 16.19 Gb (Roth *et al.* 1997). The plot of all 17-mers present in the PE300 aggregated library that were counted and the number of distinct 17-mers (*k*-mer species) for each depth from 1 to 600 shows the existence of a considerable amount of two-, three- and four-copy repeats (17-mers) in this large genome (Figure 1). The main peak at depth 91X corresponds to unique haploid sequences, while the right-most peaks at depths 182, 273, and 364 correspond to considerable fractions of multi-copy repeat sequences (Figure 1).

### Genome assembly quality

**Figure 2:**
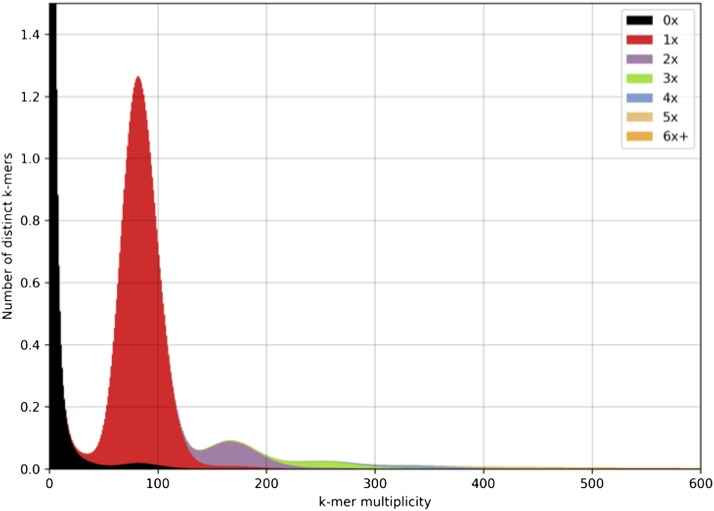
Spectra copy number in the *Abies alba* genome ABAL 1.1. Comparison between the *k*-mer (*k* = 27) spectra of paired-end (PE) 300 2 × 151 bp reads generated from the PE300 library with 300 bp long fragment inserts and the ABAL 1.1 assembly. This stacked histogram was produced with KAT (Mapleson *et al.* 2016) that shows the spectra copy number classes along the assembly.

The silver fir genome sequence presented here accounts for 18.17 Gb, with 37 million scaffolds characterized by an N50 of 14.05 Kb (Table 2). The scaffold size ranges between 106 bp and 297,427 bp with a mean size of 489.5 bp. The gaps constitute a total of 236.7 Mb and are relatively small on average (29.3 ± 46.8 bp). The assembly size is again slightly higher than the C-value of 16.19 Gb (Roth *et al.* 1997) or the *k*-mer-based estimate of 17.36 Gb (Figure 1). A comparison of frequency of spectra of 27-mers from the PE300 reads to the final assembly using KAT (Figure 2) suggests a high level of completeness: most of 27-mers in the reads belong to the haploid or main peak of the genome. Figure 2 also shows that the fractions of the genome corresponding to real 2-copy (violet) and 3-copy (green) repeats were successfully included in the assembly.

**TABLE 2:** Summary statistics for the *Abies alba* whole-genome assembly version 1.1 (ABAL 1.1) and chloroplast assembly

Genome	Feature	
**Nuclear**	Number of contigs	45,280,944
	Number of scaffolds	37,192,295
	Mean GC%	39.34
	Total length (Mb)	18,167
	Minimum scaffold length (bp)	106
	Maximum scaffold length (bp)	297,427
	Mean scaffold length (bp)	488.50
	Median scaffold length (bp)	115
	Contig N50 (bp)	2,477
	Scaffold N50 (bp)	14,051
**Chloroplast**	Total length (bp)	120,908
	Number of contigs	11
	Number of scaffolds	1
	Contig N50 (bp)	15,758

Genome completeness was estimated with three methods based on the presence of conserved genes. CEGMA estimated 81.5% completeness using 248 conserved eukaryotic genes. Using larger gene sets, BUSCO estimated a completeness of 49%, whereas mapping to the *P. taeda* transcriptome resulted in a completeness estimate of 69%. The contiguity of the silver fir assembly was also compared to those of other available conifer genome assemblies (Tree Gene Database; https://treegenesdb.org/). The scaffold N50 (scfN50) of the silver fir assembly was 14.05 Kb, almost double that of the 5.21 Kb scfN50 of the latest *P. abies* assembly (Paab1.0b) and the 6.44 Kb of the *L. sibirica* assembly (Table 3). However, it is still far below those of *P. lambertiana* (2,509.9 Kb), *P. glauca* (110.56 Kb), *P. taeda* (2,108.3 Kb) and *P. menziesii* (372.39 Kb; Table 3).

**TABLE 3:** Comparison of genome summary metrics from *Abies alba* and other sequenced conifer genomes (version numbers in parentheses)

Genome summary metric	*Abies alba (1.0)*	*Pseudotsuga menziesii (1.5)*	*Pinus taeda (2.0)*	*Pinus lambertiana (1.5)*	*Picea glauca (3.0)*	*Picea abies (1.0)*	*Larix sibirica (1.0)* *^a^*
Total length (Mb)	18,167	15,700	20,613	31,000	32,795	19,600	12,340
N50 scaffold (Kb)	14.05	372.39	2,108.3	2,509.9	110.56 34.40*^b^*	5.21	6.44
N of genes	94,205	54,830	47,602	71,117 *^c^*	102,915	70,968	49,521
N of full-length genes	50,757	20,616	NA	13,936 *^c^*	16,386 *^b^*	28,354 *^d^*	32,482
N of exons	181,168	181,475	166,465	153,111	232,182	178,049	151,838
N of introns	64,728	145,595	108,809	121,858	124,951	107,313	101,675
Mean gene length (bp)	1,190	10,510	9,066	40,820	1,330	2,427	982
Mean exon length (bp)	352	231	320	241	320	312	324
Mean intron length (bp)	311	2,301	3,004	10,164	511	1,017	353
Maximum exon length (bp)	6,300	8,037	4,946	8,003	9,568	6,068	10,268
Maximum intron length (bp)	36,015	182,831	408,800	805,500	44,116	68,269	10,154
Exons per gene	1.92	8.80	3.50	5.25	2.26	3.78	3.03
Total exonic length	6.4x10^6^	4.2x10^6^	5.3x10^6^	1.8x10^6^	7.4x10^6^	5.6x10^6^	4.9x10^6^

For the gene annotation and the definition of the “full-length genes” different approaches were used across species. The scaffold N50 (scfN50) was calculated on the unshuffled assemblies and discarding scaffolds shorter than 200 bp.

a Kuzmin *et al.* (2019).

b high confidence set (Warren *et al.* 2015; PG29 v3) and scaffold N50 calculated using sequences ≥ 500 bp: N50 is 71.5 Kb if considering both clones (WS77111)

c low-quality and high-quality gene models from *Pinus lambertiana* v.1 (Stevens *et al.* 2016), the other were calculated on *Pinus lambertiana* v1.5 (Crepeau *et al.* 2017),

d high confidence (Nystedt *et al.* 2013)

The assembly completeness is estimated to be moderately high with 81.5% of the Core Eukaryotic Genes as estimated by CEGMA, 65% of 956 plant orthologs as estimated by BUSCO and at least 69% *P. taeda* transcripts mapping to the assembly. As each of these methods are also affected by assembly fragmentation, the most likely explanation for less than ideal “completeness” is that the assembly is too fragmented for a good fraction of genes to be detected properly by the programs rather than the genes being truly missing from the assembly. While this first draft of the silver fir genome is highly fragmented, as were earlier conifer genome assemblies, likely due to the presumed density of repetitive sequences typical for plant genomes (Bennetzen and Wang 2014), it represents a very valuable reference resource to the community and can be used immediately to facilitate a broad spectrum of genetic and genomic studies in a demographic, evolutionary, and ecological context. Given the size and complexity of the silver fir genome, the low contiguity of the assembly obtained with this sequencing approach was not surprising. However, a comparison of the *k*-mer spectra of the reads used to assemble contigs (from haploid material) with their copy number in the final assembly shows that we have obtained a fairly complete assembly. In fact, the majority of the *k*-mers belonging to the main haploid peak are contained in the assembly once and only once, while the peaks of double and triple *k*-mer depth are almost purely 2-copy and 3-copy repeats. Only minor collapsing of repeats is observed. Given the haploid nature of the sample (megagametophyte), we consider these repeat tails to be a real part of the genome that will mainly contain repeats and, sometimes, partial genes, and might contain repeated genes. Therefore, these regions were included in the assembly for higher reference completeness.

### Chloroplast assembly

*De novo* chloroplast assembly, using SPADes and the *A. koreana* complete chloroplast sequence as a reference for mapping, gave an assembly totaling 123,546 bp and contig N50 of 9,211 bp. The second reference-assisted assembly with Ragout using *A. sibirica* and Gapfiller produced a single scaffold of 120,908 bp, comprised of eleven contigs (Table 2). The estimated contig N50 was 15.8 Kb. Each chloroplast has its own genome (cpDNA) that for most plants is formed by four parts: two large inverted repeats, one large single-copy and one small single-copy region. Pinaceae chloroplast genomes lack the inverted repeats. Moreover, the chloroplast genomes in Pinaceae are characterized by the presence of many small repeats and are known to vary in organization (Hipkins *et al.* 1994). The cpDNA organization in Pinaceae was investigated using the *Cedrus* cpDNA as reference, showing the presence of at least three organization types: one similar to *Cedrus* and also found in *Picea*, another similar to *Pseudotsuga*, and another similar to *Larix* (Wu *et al.* 2011). In addition to *Cedrus*/*Picea*, *Pseudotsuga* and *Larix* organizations, another form of organization was recognized in *Abies* (Tsumura *et al.* 2000). Using the DNAdiff module for genome alignment, a high collinearity was observed with the *A. koreana* and *A. sibirica* complete chloroplast sequences except for a region of ∼45 Kb that aligns in the opposite direction to *A. koreana* due to the presence of inverted repeats (Figure S3).

The size of the chloroplast assembly of silver fir was not only close to those of *A. sibirica* and *A. koreana* (Semerikova and Semerikov 2007), as expected, but also to the 124 Kb estimated in *P. abies* (Nystedt *et al.* 2013), the 124.1 Kb in *Picea sitchensis* (Coombe *et al.* 2016), the 121.3 Kb in *Abies nephrolepis* (Yi *et al.* 2015) and 122.6 Kb in *L. sibirica* (Bondar *et al.* 2019). By using Dogma 85 protein coding genes, four rRNA genes and 39 tRNA genes have been annotated. With respect to the *A. koreana* and *A. sibirica* chloroplast genomes, the *A. alba* chloroplast assembly has four duplicated tRNAs (*trn*A-UGC, *trn*I-GAU, *trn*L-UAA and *trn*V-UAC) and *trn*S-UGA has been replaced by *trn*S-CGA.

### Annotation

#### Protein-coding gene annotation:

According to the repeat annotation performed, 78% (14.25 Gb) of the genome assembly corresponds to repeats. In the non-repetitive fraction, 94,205 genes were annotated whose 98,227 transcripts encode 97,750 proteins (Table 4). However, the number of distinct genes is inflated as many partial genes have been annotated due to the large fragmentation of the assembly. Supporting this assessment, the median gene length was 558 bp, half of the genes were mono-exonic and approximately half of the annotated proteins (44,646) contained only partial ORFs (missing a start or stop codon). Of the 97,750 protein sequences, 39,420 (35.8%) were assigned functional labels, while the rest (58,327 proteins) were analyzed with BLAST, but failed to return significant hits against the RefSeq database. In total, 21,612 of the proteins with complete ORFs were functionally annotated successfully.

**TABLE 4:** Genome annotation statistics for *Abies alba* considering two types of gene models (protein coding genes and full-length genes). All statistics are given in Table S3

Features	Protein-coding genes	Full-length genes
Number of genes	94,205	50,757
Median gene length (bp)	558	804
Number of transcripts	98,227	53,487
Median transcript length (bp)	445	597
Number of exons	187,740	181,168
Coding GC content	46.4%	45.15%
Median exon length (bp)	224	237
Number of introns	89,618	64,728
Median intron length (bp)	146	145
Exons/transcript	2.00	2.32
Transcripts/gene	1.04	1.05

Two types of gene models were used to calculate the genome annotation statistics: the protein-coding genes and the full-length genes, respectively. The coding GC content was 46.4% in the protein coding genes and 45.2% in the full-length genes. While the number of exons for the protein-coding genes was 187,740 with a mean length of 327 bp, the number of introns was 89,618 (mean length: 320 bp). The number of full-length genes was 50,757 with a median gene length of 804 bp. The number of exons was 118,168 with mean length of 352 bp, the number of introns was 64,728 (mean length: 330 bp; Table 4, Table S5).

**Figure 3:**
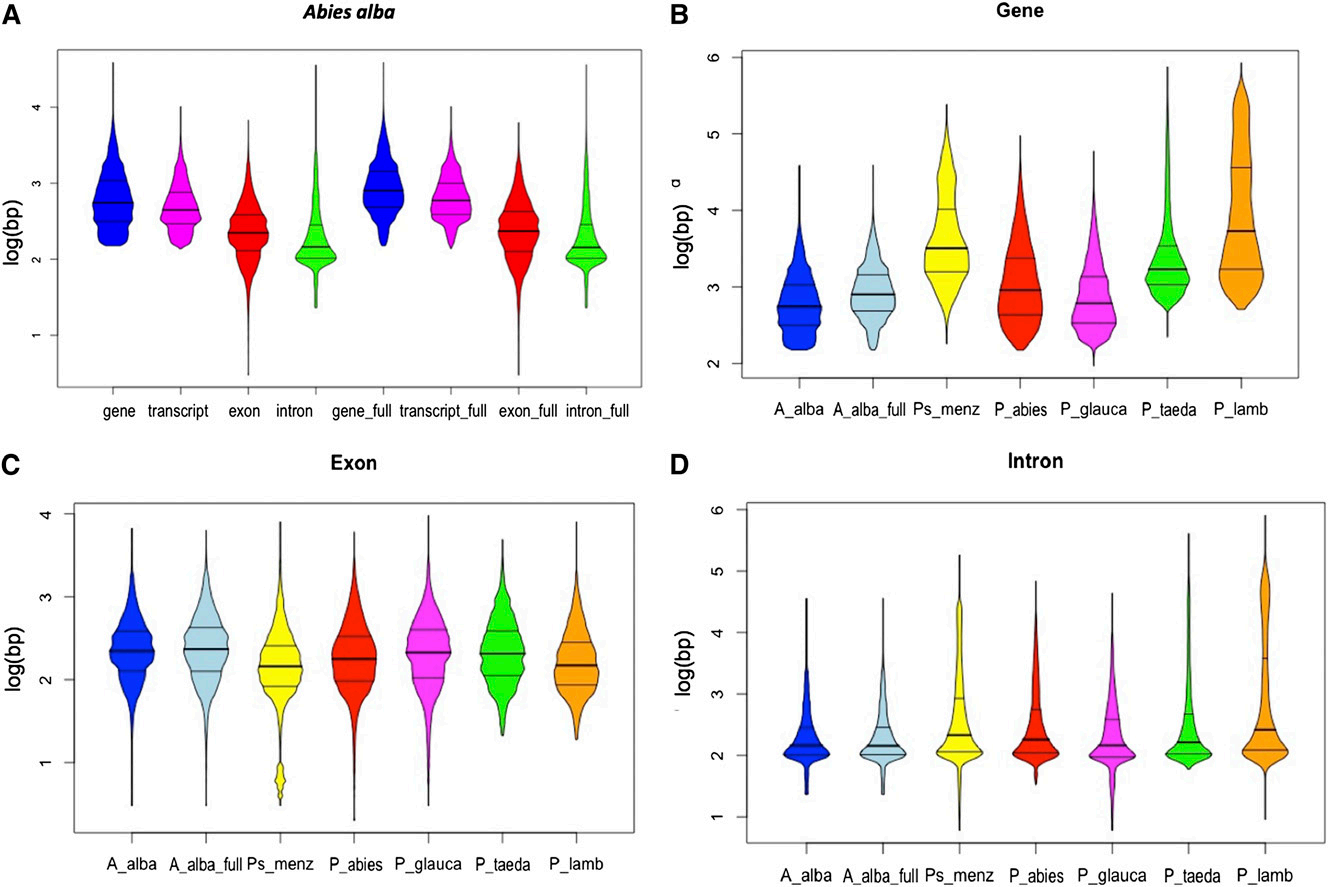
Violin plot of the distribution length of the genes, transcripts, exons and introns across the *Abies alba* (Abies_al) high-quality genes and full-length genes (indicated as “full”; A). The length was log10 transformed. Violin plot of the distribution lengths of genes (B), exons (C) and introns (D) across the *Abies alba* (A_alba) high-quality genes and full-length genes, *Pseudotsuga menziesii* (Ps_menz), *Picea abies* (P_abies), *Picea glauca* (P_glauca), *Pinus taeda* (P_taeda), *Pinus lambertiana* (P_lamb).

The distributions of the transcript, intron and exon lengths across the silver fir genome were similar in the protein coding genes and full-length genes (Figures 3A and S4). The violin plot showed a different length distribution in the low part of the violin between the two gene models, due to the lower number of short genes in the full-length gene model than in all genes.

#### Comparison With other conifers:

The comparison of silver fir genome metrics with other conifer species showed a genome size similar to *P. menziesii* and *P. abies*. Moreover, the gene numbers (94,205) without filtering for quality and completeness were similar to what was found in *P. abies* (70,968), *P. lambertiana* (71,117), and *P. glauca* (102,915), but higher than in *P. menziesii* (54,830), *P. taeda* (47,602), and *L. sibirica* (49,521). When applying a quality filter, more full-length genes (50,757) were found in silver fir than high-confidence genes in *P. lambertiana* (13,936), *P. glauca* (16,386), *P. abies* (28,354), and *P. menziesii* (20,616). The mean and maximum intron lengths were lower than in the other conifers, while mean exon size was similar to that in *P. taeda*, *P. glauca*, *P. abies* and *L. sibirica* (Table 3).

While the distributions of gene length across the genome were similar between silver fir and *P. glauca* (Figure 3B), the mean length in *P. menziesii*, *P. taeda* and *P. lambertiana* was higher than in the other conifers (Table 3). In *P. abies*, the mean gene length was close to that in silver fir, whereas its distribution range was wider (Figure S5A). The density plot using violin visualization confirmed these differences among species. In particular, the shape of this plot showed the distribution of the genes according to their lengths and highlighted the higher number of short genes in *P. abies*, *P. glauca* and silver fir than in the other conifers (Figure 3B). This comparison among the distribution of gene lengths estimated in silver fir with the values found in the assemblies of other conifers showed some interesting results. First, the genes of silver fir were on average shorter than in the other conifer species, except for *P. glauca* (1,190 bp *vs.* 1,330 bp; Warren *et al.* 2015) and *L. sibirica* (982 bp). However, this might be an effect of the sequencing strategy used and the presence of many short scaffolds in the silver fir assembly, and it will require confirmation with future improvements to the genome sequence.

Moreover, the distribution of exon and intron lengths across the silver fir genome was also compared with those found in the other fully sequenced conifers. The exon distribution was similar across species (Figure S5B), with *P. menziesii* and *P. glauca* showing a slightly lower mean value (Table 3). This was due to the short exons in *P. menziesii*, as it is visualized in the density plot (Figure 3C). The comparison of the silver fir exons in the current study with those in the other conifers showed similar values for the number, mean length and maximum length of exons, as well as the total amount of exonic sequence (63.7 Mb *vs.* the mean of 50.8 Mb for all compared annotations). This result confirmed that the number and the length of exons are well conserved across species (Stival Sena *et al.* 2014). The average number of exons per gene was less conserved and the smallest in silver fir (1.92) compared to all other conifers (2.26-8.80). The mean number of exons per gene averaged for all seven species was 4.08, which is very close to the value of 3.66 predicted for species such as conifers (Table 2 in Koralewski and Krutovsky 2011). Given that the average amount of exonic sequence in the conifer genomes analyzed here is only 50.8 Mb, the differences in genome size among conifers are presumably due in large part to the large fraction of repetitive sequences they contain (Morse *et al.* 2009; Wegrzyn *et al.* 2013, 2014). Moreover, one of the major components of plant genomes are the transposable elements, which may also affect the evolution of the intron size (Kumar and Bennetzen 1999).

Silver fir intron and exon statistics were compared to *P. menziesii*, which was sequenced, assembled and annotated using a similar approach (Table S5). For *P. menziesii*, the genes were classified into two categories that were based on gene quality and completeness (high-quality and high-quality full-length) and the counts were calculated for both categories. While the numbers of exons and their means were similar in the two species (187,740 for the protein-coding gene model in silver fir and 181,475 for the high-quality gene model in *P. menziesii*), a lower number of introns with a lower mean size was found in silver fir than in *P. menziesii* (89,618 and 145,595, respectively).

The distribution of intron lengths was similar across all species (Figure 3D), with silver fir showing a narrower distribution range than the other conifer species (Figure S5C). Although intron size has been positively correlated with genome size across eukaryotes (Vinogradov 1999), this trend is not a rule for seed plants (Wan *et al.* 2018). Previous studies have reported larger intron sizes in conifers than in angiosperms (Nystedt *et al.* 2013; Neale *et al.* 2014; Guan *et al.* 2016; Stival Sena *et al.* 2014). This difference is probably related to the high percentage of repetitive sequences, which are the major component of all gymnosperm genomes sequenced to date. Across gymnosperms, *Ginkgo biloba* has longer introns (Guan *et al.* 2016) than *P. taeda*, but a smaller genome. When comparing the distribution of intron lengths across genomes in several conifers, we found a similar distribution and average between silver fir and *P. glauca* (311 bp *vs.* 511 bp), with the genome size of the latter being almost double (33 Gb) that of silver fir. Moreover, the smallest both mean and maximum intron lengths were observed in *A. alba* and *L. sibirica* that have also the smallest genome sizes, 16.19 Gb (Roth *et al.* 1997) and 12.03 Gb (Ohri and Khoshoo 1986), respectively.

Another aspect related to intron length is the suggestion that the expansion of introns occurred early in conifer evolution (Nystedt *et al.* 2013). This hypothesis was confirmed by the comparison between orthologous introns of *P. taeda* and *G. biloba* that showed a high content of repeats in long introns in both species (Wan *et al.* 2018). In addition, our analysis showed that the maximum intron lengths occur in *P. taeda* and *P. lambertiana*, and their mean intron length was higher than in other conifer species. The geological timescale calculated for the Pinaceae showed that *Pinus* is the oldest genus across the Pinaceae, since its presence was confirmed starting from the Early Cretaceous (Wang *et al.* 2000). The genus *Abies* should be closer to *Pseudotsuga* than to *Picea* and *Pinus* (Wang *et al.* 2000). Nevertheless, likely due to the high fragmentation of the silver fir genome sequence reported here, the estimated maximum intron length in *A. alba* was only half of that estimated for *P. menziesii*.

The input file accounted for 462,216 GO terms that were mapped to the slim2 classification list categories. The total count (Table S6A) was 27,723 terms corresponding to 32,272 genes, of which 12,221 unique terms belonged to at least one of the 110 slim2 classes. The 462,216 GO terms were mapped to the myclass2 classification list categories. The total count (Table S6B) was 31,839 terms corresponding to 32,275 genes, of which 12,361 unique terms belonged to at least one of the 162 myclass2 classes.

In both classification lists, the main categories were metabolism (11.1% and 9.7% for slim2 and myclass2, respectively), catalytic activity (7.7%, 6.7%), cell (4.7%, 4.1%) and cell organization (4.3%, 3.7%; Table S5, Figure S6A and Figure S6B).

### Conclusions and Perspectives

Here, we present a draft version of the silver fir genome, which represents a first step toward the full deciphering of this giga-genome in its entire complexity. This research was accomplished by the Alpine Forest Genomics Network (AForGeN). The approach applied in this project could serve as a model for sequencing additional plant and animal genomes. The genome sequencing was financed by a bottom-up approach among partners, which could possibly be a profitable strategy for many (plant) genome-sequencing initiatives in the future (Twyford 2018).

Future research projects could utilize the draft silver fir genome as a reference to re-sequence a diverse panel of trees from contrasting environments and to develop a genotyping array with thousands of single-nucleotide polymorphisms (SNP). Such SNP resources will be useful in many types of demographic studies and, along with the gene annotation presented here, will enable genomic studies and experiments aimed at discovering those genes that are relevant for particular traits (*e.g.*, related to growth) and adaptive responses (*e.g.*, drought tolerance).
